# The Diabetes Camp Paradox: An Evidence Gap Map of 279 Publications and the Anatomy of Known Unknowns

**DOI:** 10.1111/hex.70833

**Published:** 2026-08-22

**Authors:** M. Nurullah Kurutkan, Ilknur Arslanoğlu

**Affiliations:** ^1^ Department of Health Management, Faculty of Business Düzce University Düzce Türkiye; ^2^ Department of Pediatric Endocrinology, Faculty of Medicine Düzce University Düzce Türkiye

**Keywords:** camps, cost‐effectiveness, diabetes mellitus, evidence gaps, long‐term care, nursing, paediatric nursing, patient education, scoping review, self‐management

## Abstract

**Aim:**

To map the diabetes camp literature across five epistemic layers and to construct an operationalised evidence gap matrix identifying structural knowledge gaps relevant to nursing practice, education and policy.

**Design:**

Scoping review reported in accordance with the PRISMA‐ScR guideline.

**Data Sources:**

Web of Science Core Collection, Scopus, OVID/MEDLINE and PubMed, searched on 19 June 2026 without date restriction (results spanned 1931–2026).

**Review Methods:**

A title‐field search (‘diabet*’ AND ‘camp’) across four databases yielded 1591 records that, after homonym screening, cross‐database deduplication, publication‐type and English‐language filtering, and a supplementary PubMed search, produced a deduplicated evidence map of 279 records. Two reviewers independently double‐coded the analytic set; inter‐rater reliability was almost perfect (Cohen's kappa 0.955 for outcome direction and 0.960 for study design). The 199 records carrying an extractable abstract formed the analytic set and were coded across four dimensions (outcome direction, study design, follow‐up duration and thematic area); proportions are reported with Wilson 95% confidence intervals.

**Results:**

Of the 199 analytic records, 104 (52.3%) reported a measured directional outcome; of these, 90.4% documented benefit in at least one dimension, but 32.7% contained mixed or non‐significant sub‐outcomes and only 57.7% were entirely positive. Only 12 records (6.0%) reported follow‐up of 6 months or longer, and post‐camp glycaemic deterioration was documented consistently across the longitudinal evidence. The evidence base was predominantly descriptive (51.8%), with only 6.5% employing a controlled experimental design; just 9 records (4.5%) combined an analytic‐or‐stronger design, follow‐up of at least 6 months and a measured outcome. No study provided cost‐effectiveness analysis, per‐camp unit cost data or comparative evidence on physical versus virtual delivery models.

**Conclusion:**

The diabetes camp literature is structurally incomplete: benefit is selective, temporally fragile, socially inequitable and entirely uncosted. Resolving this paradox requires temporal expansion (12‐month follow‐up), dimensional expansion (multidimensional outcome packages) and structural expansion (cost‐effectiveness analysis and delivery‐model comparison).

**Impact:**

This review identifies actionable evidence gaps for paediatric, public health and school nurses involved in camp‐based diabetes self‐management education, informing nurse‐led longitudinal follow‐up, equitable access strategies and economic accountability in nursing‐delivered camp interventions.

**Patient or Public Contribution:**

The first author is fathering a child with autism, and he is focusing on rationalistic design of health services during his career. The co‐author is the head of a nonprofit association established in 2010, the mission of which is advocating, supporting, educating and motivating children with diabetes and their families. Its board is made of three mothers of children with diabetes, one young adult with diabetes, one social worker who has been part of the paediatric diabetes team for 20 years and one diabetes specialist nurse. This association conducts numerous diabetes camps every year and is pioneering camp activities in the country by far. A big part of these camps is conducted in limited resource settings, which was reviewed in an article published in *Diabetes Voice*, the official journal of the International Diabetes Federation. The article we submit now is motivated by these activities.

## 1 Introduction

Diabetes camps are structured, predominantly nurse‐coordinated interventions that have integrated education, clinical care, psychosocial support and peer interaction for individuals with diabetes since the 1920s [1]. Rosenbloom [2] argued that camps offer unique advantages that cannot be replicated in clinical settings; Maslow and Lobato [3] compiled histories, safety records and outcomes, and Barone et al. [4] evaluated the question ‘are diabetes camps effective?’ from an interrogative perspective using data from all continents.

However, systematic inconsistencies lie beneath the general optimism of the literature. Wang et al. [5], while reporting positive glycaemic outcomes in their own study, noted that improvement could not be consistently demonstrated in the prior literature. Hasan et al. [6], in their meta‐analysis, found the glycated haemoglobin reduction significant but reported that quality‐of‐life and anxiety improvements failed to reach significance. Scaramuzza et al. [7] directly posed the question: ‘Does educational camp always benefit?’ These conflicting signals point to what can be termed the ‘Diabetes Camp Paradox’: camps mostly provide benefit; however, the scope, sustainability, variability by target population and economic dimension of this benefit are insufficiently known.

This study aims to deconstruct this paradox by analysing a four‐database evidence map of 279 records, of which 199 with extractable abstracts form the analytic set, through five epistemic layers: (1) what do we know, (2) how reliably do we know it, (3) for how long do we know it, (4) for whom do we know it and (5) what do we not know. This five‐layered approach is intended to map the literature not only thematically but across epistemic depth levels of direct relevance to nursing practice, education and policy.

## 2 Background

Diabetes camps occupy a unique position in nursing‐led chronic‐disease management. From the earliest North American camps in the 1920s, registered nurses have been the principal coordinators of insulin administration, hypoglycaemia surveillance, blood‐glucose monitoring and patient education at these settings. Contemporary camps continue to be staffed predominantly by paediatric, public health and school nurses, complemented by nursing students serving as counsellors—a workforce structure with documented developmental impact on student attitudes, self‐efficacy and clinical confidence [8, 9, 10].

Within Donabedian's structure–process–outcome framework, the camp environment functions as a high‐density nursing intervention: structurally, it concentrates nurse‐to‐patient ratios well beyond outpatient norms; procedurally, it integrates education, surveillance and psychosocial support within a closed residential cycle, and at the outcome level, it has been associated with short‐term improvements in glycaemic control and self‐management knowledge. Despite this nursing centrality, evaluative scholarship has been published largely in endocrinology and paediatrics outlets rather than in nursing journals, and global‐level guidance from nursing professional bodies—such as the International Council of Nurses, the Royal College of Nursing and the Association of Diabetes Care & Education Specialists—on camp‐based programmes remains comparatively underdeveloped. This nursing‐visibility gap motivates the present scoping review: to deliver an evidence map that is methodologically rigorous and explicitly oriented to the planning, equity and accountability questions facing nurses who design, deliver and lead diabetes camps internationally.

## 3 Methods

### 3.1 Reporting Method

This scoping review was conducted and reported in accordance with the PRISMA‐ScR (Preferred Reporting Items for Systematic Reviews and Meta‐Analyses extension for Scoping Reviews) reporting guideline [11]. A completed 22‐item PRISMA‐ScR checklist accompanies this submission as Supporting File S1. The review protocol was prospectively deposited in a public repository before screening commenced (anonymised repository link to be reinstated post‐acceptance).

### 3.2 Search Strategy and Data Source

Searches were conducted on 19 June 2026 in four bibliographic databases: Web of Science Core Collection, Scopus, OVID/MEDLINE and PubMed. For Web of Science, Scopus and OVID/MEDLINE, a title‐field truncated query combining the disease and setting terms (‘diabet*’ AND ‘camp’) was used (Web of Science: TI = (diabet* AND camp); Scopus: TITLE(diabet* AND camp); OVID/MEDLINE: (diabet* and camp).ti.), yielding 1591 records (Web of Science 750, Scopus 508 and OVID/MEDLINE 333). Because the unfiltered PubMed title query returned 4555 records dominated by the cyclic‐AMP homonym, PubMed was searched with an a priori filtered strategy that excluded ‘cyclic AMP’/‘cAMP’ and ‘refugee’ at the query level and was added as a supplementary source. Embase was not searched because the authors' institutions hold no Embase subscription; this is acknowledged as a limitation. The full per‐database strategies are provided in Supporting File S2.

### 3.3 Eligibility Criteria

Inclusion criteria: (a) publications directly addressing camp interventions for individuals with diabetes, research conducted in camp settings or camp organisation; (b) publications in English, and (c) peer‐reviewed journal articles, early‐access articles, systematic reviews and meta‐analyses.

Exclusion criteria: (a) publications using the word ‘camp’ outside the context of diabetes camps (e.g., ‘CAMP protein’, ‘Chemical Abstracts Machine Processing’); (b) epidemiological studies reporting diabetes prevalence in refugee camps without involving camp interventions, and (c) chronic‐disease camps other than diabetes.

### 3.4 Screening Process

**Figure 1:**
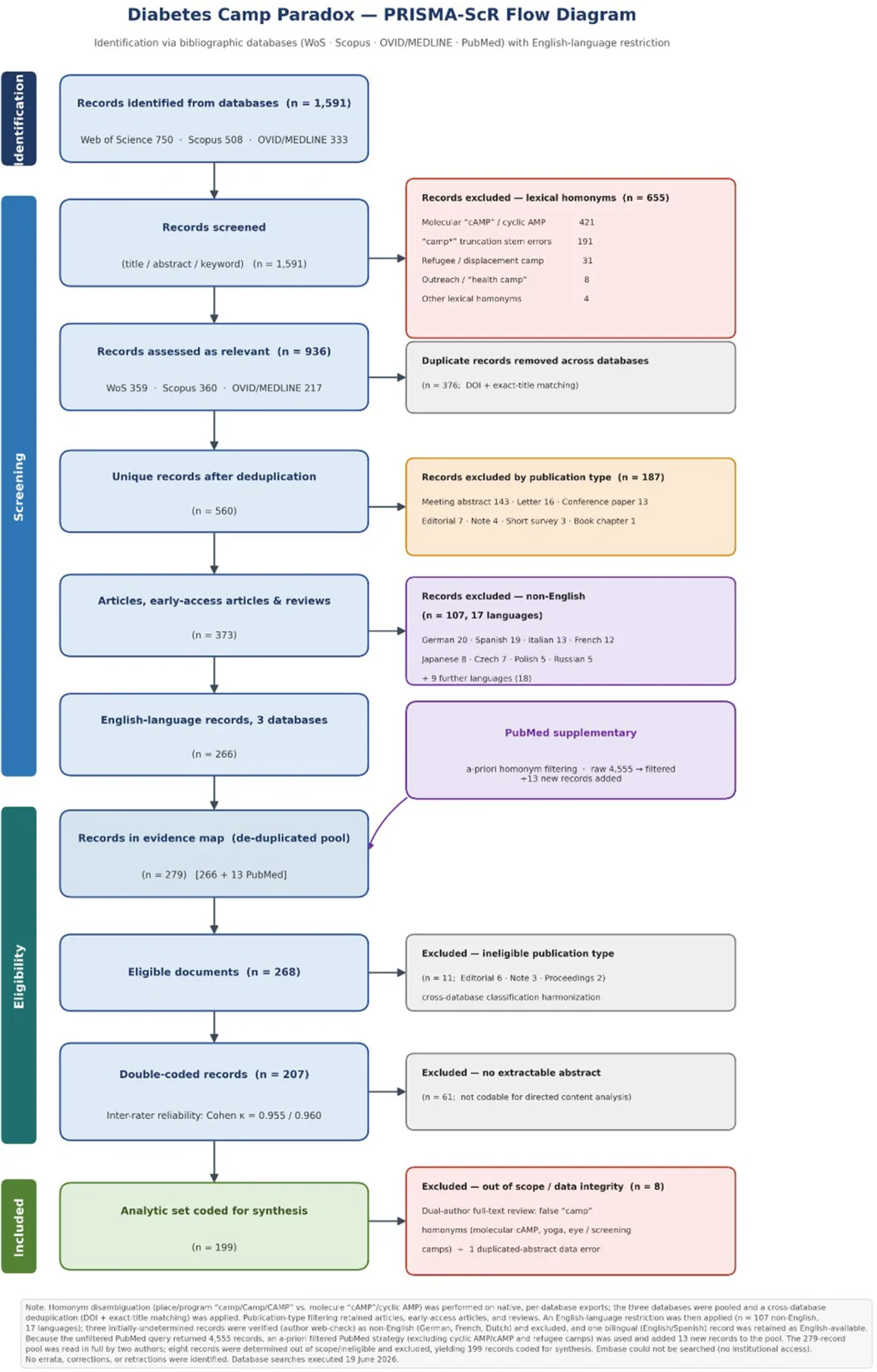
PRISMA‐ScR flow diagram of the search, screening and charting process. A title‐field search across four databases (Web of Science 750, Scopus 508 and OVID/MEDLINE 333) yielded 1591 records; after homonym screening, deduplication, publication‐type and English‐language filtering, and a supplementary PubMed search (13 new records), a deduplicated evidence map of 279 records was obtained, from which 199 records with extractable abstracts formed the analytic set.

The pooled 1591 records were screened for lexical relevance against native, per‐database exports; 655 homonym records were removed (molecular cyclic AMP/cAMP, ‘camp*’ truncation stem errors, refugee or displacement camps, outreach or health camps and other lexical homonyms). Cross‐database deduplication (DOI plus exact‐title matching) removed 376 duplicates, leaving 560 unique records. Publication‐type filtering retained journal articles, early‐access articles and reviews and removed 187 records; an English‐language restriction then removed 107 non‐English records across 17 languages, leaving 266 records. The supplementary PubMed search added 13 new records, producing a deduplicated evidence map of 279 records. The full text of all 279 records was independently read by two reviewers (a senior academic in health management and a master's‐level researcher); eight records were determined to be out of scope or to contain a data‐integrity error and were excluded by consensus, and the 199 records carrying an extractable abstract formed the analytic set. The two interpretively demanding dimensions, outcome direction and study design, were independently double‐coded blind; inter‐rater agreement was almost perfect (Cohen's kappa 0.955, 95% CI 0.920–0.991, for outcome direction; 0.960, 95% CI 0.929–0.992, for study design), and all disagreements were resolved by consensus. The screening and charting cascade is summarised in Figure 1. Year distribution of the analytic set: pre‐2000 *n* = 51; 2000–2009 *n* = 32; 2010–2019 *n* = 62, and 2020–2026 *n* = 54. Database provenance was recorded for every record and is presented as a four‐set Venn diagram of the analytic set (Supporting File S3).

### 3.5 Coding Protocol and Analytical Framework

The 199 records with extractable abstracts were coded across four dimensions.

#### 3.5.1 Research Design

Studies were classified into the following design categories: descriptive reports, prospective cohort studies, randomised controlled trials (RCTs) or crossover trials, qualitative studies, retrospective analyses, pilot or feasibility studies and systematic reviews or meta‐analyses (distribution of included studies reported in Section 6.2).

#### 3.5.2 Outcome Direction

Classified into three categories through linguistic analysis of outcome statements in abstract texts: (a) positive—reporting statistically significant improvement in all primary outcomes; (b) mixed—reporting significant improvement in at least one primary outcome while others failed to reach significance or contained negative results, and (c) negative/null—no significant improvement detected in any primary outcome. Outcome‐direction coding was applied only to the 104 records reporting a measured outcome; the remaining 95 records did not report a directional outcome.

#### 3.5.3 Follow‐Up Duration

Stratified as camp‐only (≤ 1 week), short‐term (1–4 weeks), medium‐term (1–6 months) and long‐term (≥ 6 months).

#### 3.5.4 Thematic Area

Determined through keyword frequency analysis and pattern matching in title, abstract and keyword texts.

### 3.6 Processing of Title‐Only Records

Records without an extractable abstract were not eligible for the analytic set. Because outcome direction, study design and follow‐up cannot be coded reliably from a title alone, such records were excluded from the analytic set (*n* = 199) and from all four‐dimension coding rather than being assigned partial codes; they remain part of the broader deduplicated evidence map (*n* = 279) only for describing the breadth of the field. This separation of the evidence map from the analytic set replaces the earlier title‐level grey‐zone approach and ensures that every coded proportion derives from an abstract‐bearing record.

### 3.7 Operational Criteria for the Evidence Gap Matrix

Three levels of completeness in the matrix were determined by the following rules. ● Strong: at least 10 studies available in the relevant area, including at least 3 prospective/RCT‐designed studies or 1 meta‐analysis. ◑ Partial: 3–9 studies available in the relevant area, and/or existing studies are predominantly descriptive in design or results are inconsistent. ○ Empty: 0–2 studies available in the relevant area, or no direct studies exist (only indirect references).

These completeness rules also define how evidence gaps were identified: a gap corresponds to a matrix cell that is empty or only partially populated under the rules above. Consequently, the gaps reported in Section 5.5 are an output of the coded evidence rather than an a priori selection by the authors, and a reader applying the same rules to the analytic set would identify the same cells.

## 4 Results

### 4.1 Layer 1: Existing Evidence Base

Of the 104 analytic records reporting a measured directional outcome, 90.4% (*n* = 94) reported benefit in at least one dimension. The evidence base spans seven core thematic areas. The metabolic/glycaemic theme is the most dominant (56 records); in this area, multicentre randomised crossover trials [12] and systematic reviews [6] provide the strongest evidence. Prospective cohort studies [13, 14] and descriptive reports [15, 16] form additional evidence layers.

**Figure 2:**
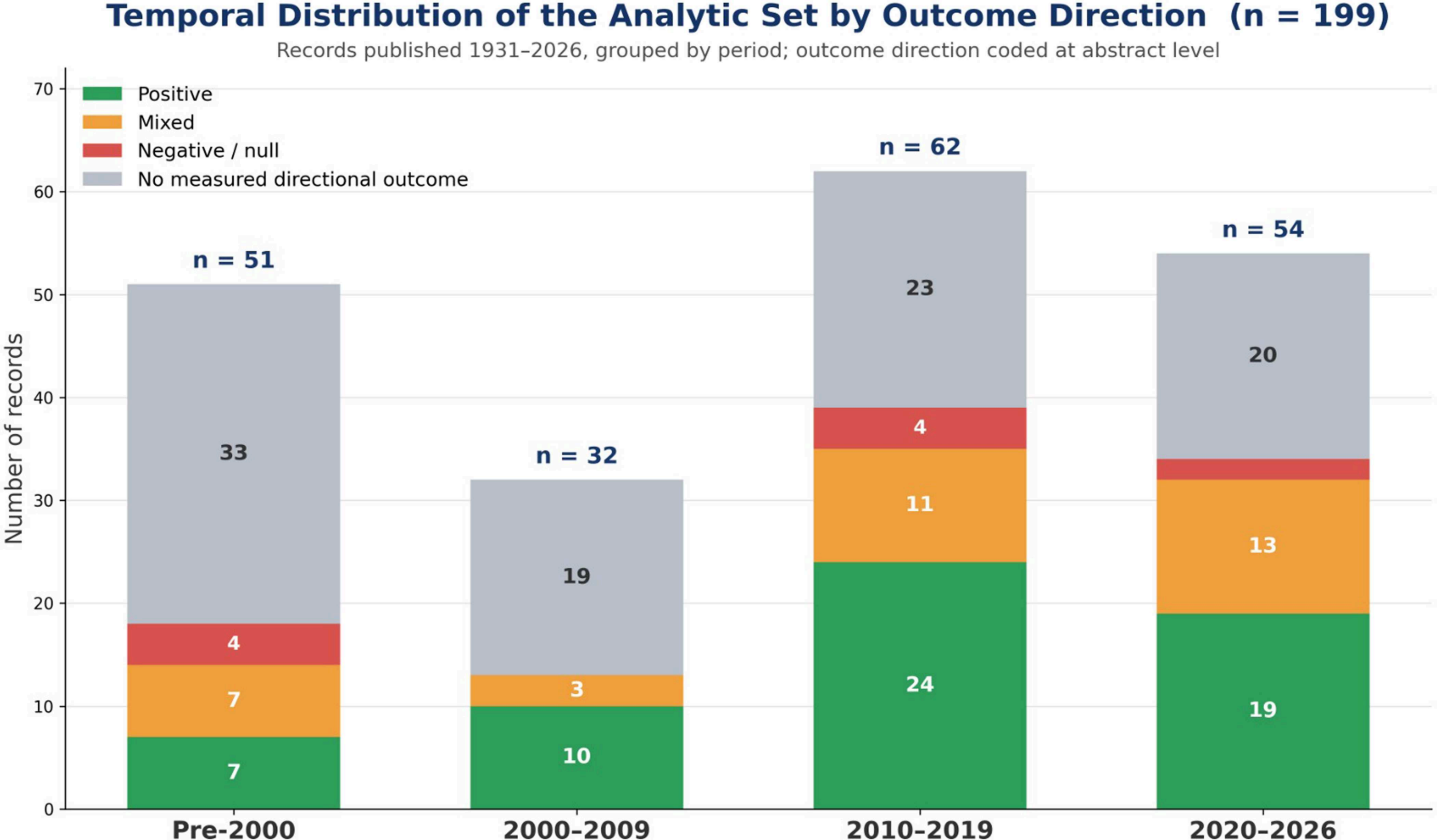
Temporal distribution of the 199 analytic records by outcome direction (1931–2026). Records reporting a measured directional outcome are categorised as positive, mixed or negative/null; records without a measured directional outcome are shown in grey. Year distribution: pre‐2000 *n* = 51; 2000–2009 *n* = 32; 2010–2019 *n* = 62, and 2020–2026 *n* = 54.

**Figure 3:**
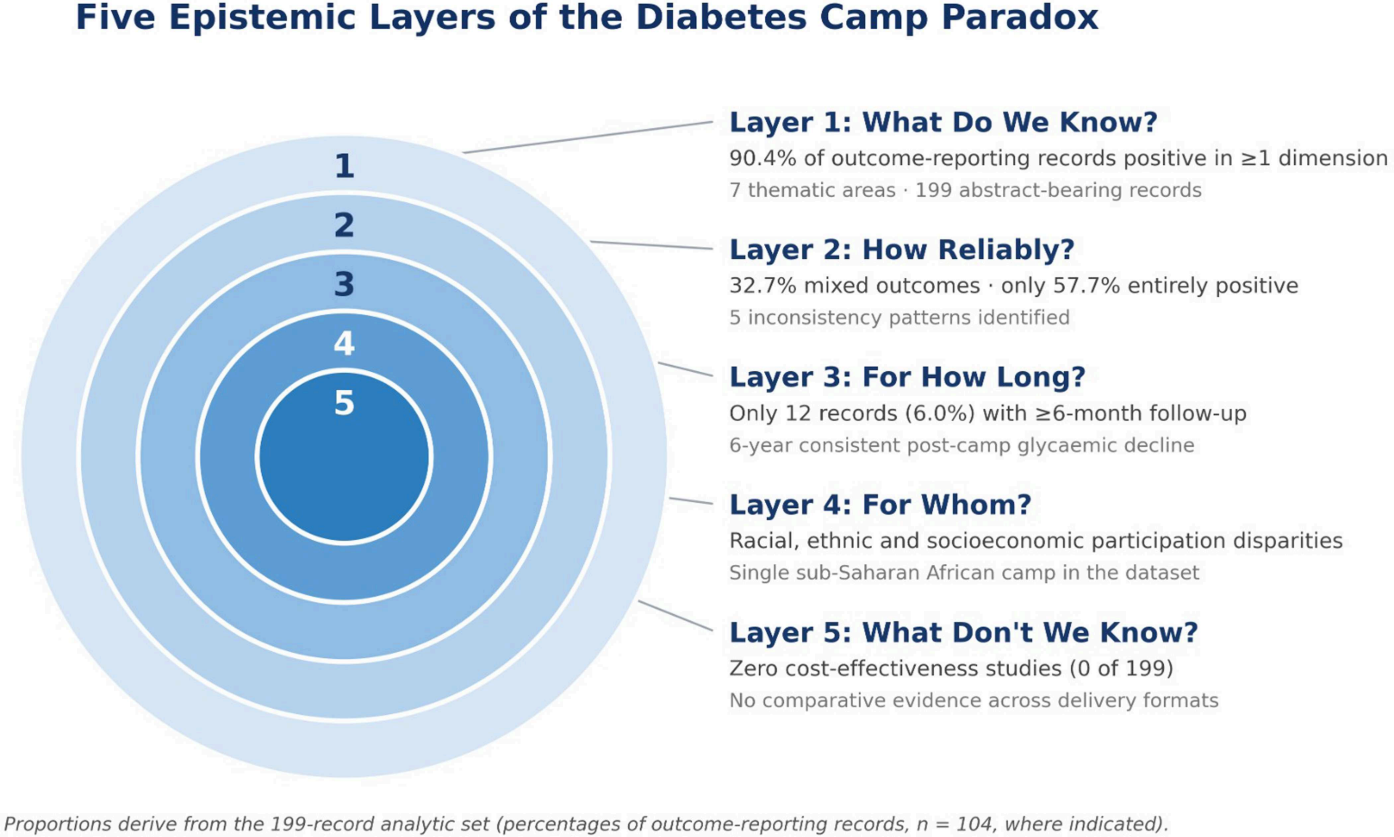
Conceptual diagram of the five epistemic layers structuring the analysis: (1) existing evidence base, (2) evidence reliability, (3) temporal sustainability, (4) distribution of benefit and access equity and (5) critical evidence gaps.

In the psychosocial theme (38 records), the evidence base is more heterogeneous: RCTs [17], prospective follow‐up studies [18] and descriptive cross‐sectional designs [8] coexist. The technology theme (27 records) includes studies in which camp settings served as platforms for technology validation; Fegan‐Bohm et al. [1] noted that camps serve as a setting for research questions unrelated to the camp itself. The analytic set also spans organisational/safety (33 records), education/self‐care (24 records), professional learning for occupational groups including pharmacy and nursing students (14 records [9, 10, 19]), and access/equity (4 records) (Figures 2 and 3).

### 4.2 Layer 2: Evidence Reliability

Of the 104 records reporting a measured outcome, 57.7% (*n* = 60) were entirely positive, 32.7% (*n* = 34) reported mixed results and 9.6% (*n* = 10) reported entirely negative or null outcomes. The substantial proportion of mixed results (32.7%) constitutes an important cautionary signal regarding the reliability of the literature. These mixed results manifest in five distinct patterns.

a. **Co‐occurrence of metabolic improvement and psychosocial stagnation.** Santiprabhob et al. [20] reported improvement in knowledge while detecting no change in quality of life and glycaemic control. Hasan et al. [6] reported that quality‐of‐life and anxiety improvements yielded *p* > 0.05.
b. **Selectivity among psychosocial dimensions.** Weissberg‐Benchell et al. [21] found improvement in distress while detecting no change in strengths. Nicholl et al. [22] observed no psychosocial changes in counsellors.
c. **Multidirectional emotional outcomes.** Rabbone et al. [23] reported improvement in positive emotions but significant worsening in mixed emotions.
d. **Technology interventions failing to demonstrate expected superiority.** Ly et al. [24] found no difference with the Medtronic hybrid closed‐loop system, and Palisaitis et al. [25] detected no time‐in‐range difference with the learning algorithm.
e. **Outcomes varying by target population.** Dyck et al. [26] found significant improvement in health professionals but not in participants with diabetes.

### 4.3 Layer 3: Temporal Sustainability

The vast majority of the literature reports only outcomes during or immediately after camp; only 12 records (6.0%) reported follow‐up of 6 months or longer, and 34.7% reported only in‐camp (1 week or less) observation. The majority of the long‐term studies document the fading of metabolic gains. The strongest longitudinal evidence belongs to Sotiriou et al. [27]: post‐camp glycaemic deterioration recurred consistently over six consecutive years. Karagüzel et al. [13] showed that body‐mass index and insulin dose returned to pre‐camp levels at 12‐month follow‐up. Santiprabhob et al. [28] explicitly characterised the metabolic effect as short‐lived.

A partial exception is models involving technology initiation. Rabbone et al. [23] demonstrated persistent time‐in‐range improvement at 6 months; however, this effect may be attributable to the closed‐loop technology itself rather than the camp intervention. Redon et al. [29] provided 10‐year insulin‐regimen trend data, but this study represents cross‐sectional comparison of successive cohorts rather than individual longitudinal follow‐up. Tomasi and Miselli [30] pointed to the need for reinforcement of post‐camp education at the earliest period of the literature.

### 4.4 Layer 4: Distribution of Benefit and Access Equity

Valenzuela et al. [31] documented racial and ethnic participation disparities with a strong sample size. Ferrari et al. [32] were the first to systematically examine participation barriers. Hull and Indyk [33] addressed rural/urban disparities, and Gumkowski et al. [34] provided a comprehensive assessment of barriers. Dehayem et al. [35] reported a pioneering camp experience in sub‐Saharan Africa and emphasised that the metabolic effect was ‘very little known’ in this region. It should be noted that a significant portion of the evidence in the access‐equity domain relies on conference proceedings, and full‐text peer‐reviewed articles are limited. These conference abstracts are cited as contextual evidence only and are not part of the 199‐record analytic set.

### 4.5 Layer 5: Critical Evidence Gaps

The most important layer of the literature is the structural evidence gaps that feed the paradox. Three structural gaps emerged from the evidence map and are graded below as critical or significant according to whether the absence carries an identifiable risk.

To avoid imposing prior expectations, the gaps reported in this layer were not selected a priori; they were derived from the evidence map itself. Applying the pre‐specified completeness rule (Section 4.7) to the 199‐record analytic set, a cell was flagged as a gap when it was empty or only partially populated: the cost‐evidence column is empty for every thematic area (0 of 199 records), durable follow‐up of at least 6 months is present in only 6.0% of records and controlled intervention evidence is confined to two domains. The gaps below are therefore the cells that the coded evidence leaves structurally unfilled rather than concerns external to the data. We reserve the term critical for absences that carry an identifiable risk to programme sustainability, equitable access or patient safety, and label the remainder significant or desirable.

#### 4.5.1 First Critical Gap: Absence of Financial Analysis

In the 199‐record analytic set, and across the broader 279‐record evidence map, no study was identified that provides direct cost‐effectiveness analysis, per‐camp unit‐cost calculation, budget‐structure definition or revenue model. The financial dimension becomes visible only indirectly, through barrier research [32], inequality documentation [31], volunteer dependency [36] and cost reduction implied by virtual alternatives [37].

The absence of economic evidence is characterised here as critical because the dataset itself documents the conditions that make funding fragile: camps depend heavily on volunteer clinical labour [36] and face documented participation barriers and inequities [31, 32]. Where programmes are sustained by philanthropy, corporate sponsorship or community benefit, as is common in high‐income settings, cost‐effectiveness may not be the primary driver of support; even so, the absence of any per‐camp cost, budget or cost‐effectiveness data carries three concrete risks. First, without economic evidence, camps cannot be compared with competing interventions for inclusion in health‐system budgets or reimbursement, leaving them dependent on discretionary funding cycles. Second, equitable scaling is constrained: the single sub‐Saharan African camp in the dataset reported that the metabolic effect was very little known in that setting [35], and lower resource regions cannot plan or justify provision without unit‐cost models. Third, value‐for‐money evidence is a precondition for converting episodic, donor‐dependent camps into sustained, nurse‐led services. The gap is therefore critical in the specific sense of an identifiable threat to sustainability and equity rather than a merely desirable addition.

#### 4.5.2 Second Critical Gap: Lack of Universal Operational Guidance

Position statements [38], safety protocols [39, 40] and partnership models [41] exist. However, Chang and Ogihara [42] reported that formal guidelines for activity design and sustainable development are lacking.

#### 4.5.3 Third Gap (Significant): Absence of Delivery‐Model Comparison

The COVID‐19 pandemic popularised virtual camps [37, 43, 44]. However, no study was identified that directly compares physical and virtual models in terms of clinical effectiveness, psychosocial outcomes and cost.

We acknowledge that fully virtual camps, which expanded during the COVID‐19 pandemic, may not persist as a mainstream delivery model, and we therefore do not rest this gap on the durability of virtual provision. The underlying gap is the broader absence of comparative effectiveness evidence across delivery formats, including the intensity, dosage and duration of in‐person camps and digitally augmented or hybrid post‐camp follow‐up, which is the mechanism most plausibly linked to the loss of metabolic gains documented in Layer 3. Because this absence does not by itself carry an immediate risk, we characterise it as a significant and answerable gap rather than a critical one.

### 4.6 Operationalised Evidence Gap Matrix

The following matrix (Table 1) was constructed at the intersection of six outcome areas and four evidence dimensions using the operational criteria defined in Section 4.7 (●: ≥ 10 studies + ≥ 3 prospective/RCT or 1 meta‐analysis; ◑: 3–9 studies or predominantly descriptive/inconsistent, and ○: 0–2 studies or only indirect references). Numbers in parentheses indicate the number of studies directly contributing to each cell. The matrix is read row by row: each row corresponds to one of the six outcome areas and each column to one of the four evidence dimensions, so the symbol–count pair in each cell shows at a glance both the strength grading and the volume of evidence at that intersection. For example, the Metabolic row combines strong positive evidence (●, 31 studies) with an entirely empty cost column (○, 0 studies).

**Table 1:** Operationalised evidence gap matrix of the analytic set (*n* = 199).

Outcome area	Positive evidence	Intervention evidence	Long‐term evidence	Cost evidence
Metabolic	● (31)	◑ (4)	◑ (5)	○ (0)
Psychosocial	● (23)	○ (1)	◑ (3)	○ (0)
Education/self‐care	● (14)	○ (2)	○ (2)	○ (0)
Technology	● (16)	◑ (6)	○ (1)	○ (0)
Organisational	○ (0)	○ (0)	○ (0)	○ (0)
Financial	○ (0)	○ (0)	○ (0)	○ (0)

*Note:* Numbers in parentheses represent the number of studies directly contributing to the relevant cell. Symbols: ● = strong, ◑ = partial and ○ = empty. Classification criteria are defined in Section 4.7.

**Figure 4:**
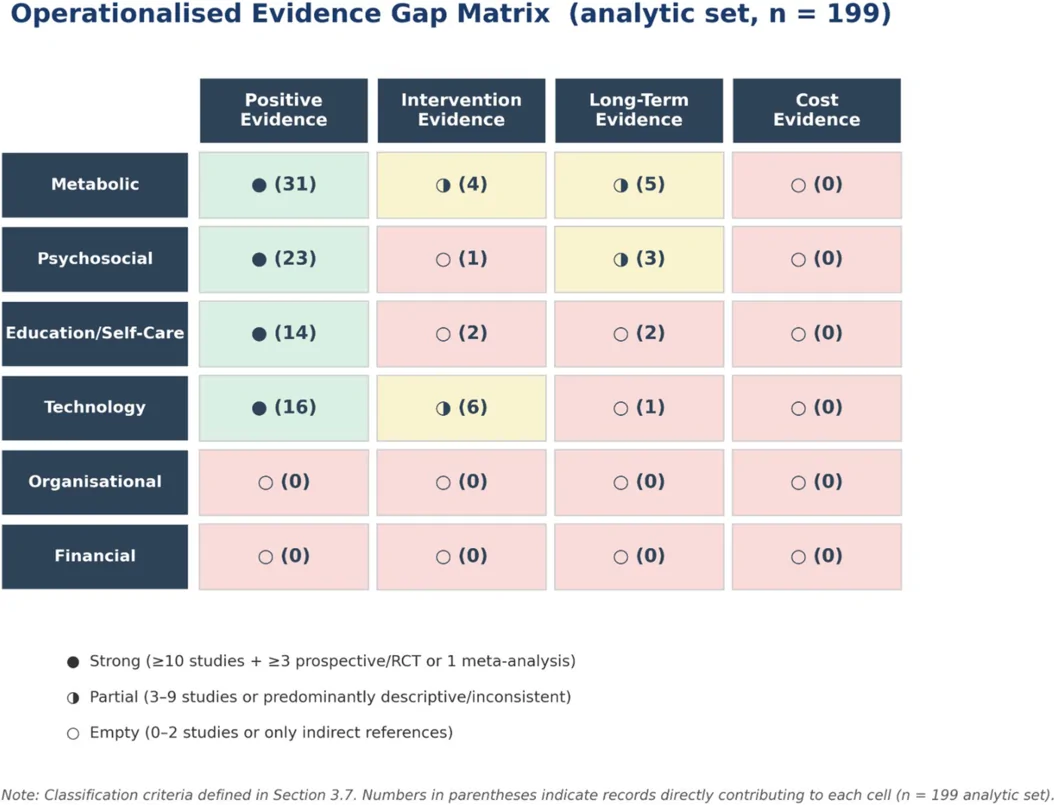
Operationalised evidence gap matrix at the intersection of six outcome areas and four evidence dimensions. Classification criteria: ● strong (≥ 10 studies including ≥ 3 prospective/RCT designs or 1 meta‐analysis); ◑ partial (3–9 studies or predominantly descriptive/inconsistent), and ○ empty (0–2 studies or only indirect references). Numbers in parentheses indicate studies directly contributing to each cell.

The matrix demonstrates the structural imbalance of the literature. The positive‐evidence column is strong in the metabolic, psychosocial, educational and technological areas, whereas the cost‐evidence column is empty across every row. The long‐term‐evidence column is only partially complete in the metabolic and psychosocial areas and is empty elsewhere, and controlled intervention evidence is concentrated in the metabolic and technology areas. This pattern indicates that the literature documents short‐term benefit while leaving durability, cost and equity structurally under‐evidenced. In practical terms, moving from left to right along any row of Table 1 traces the transition from ‘whether camps help’ to ‘how, for how long and at what cost they help’, and it is precisely along this axis that the coded record empties (Figure 4).

## 5 Discussion

### 5.1 Structural Root Cause of the Paradox

The root cause of the Diabetes Camp Paradox stems from the literature's imbalanced treatment of three temporal and structural dimensions. The vast majority of publications report short‐term outcomes during camp, neglecting sustainability after camp and how the camp itself is structured and financed. The literature has broadly answered the question ‘Does it work?’ (yes, mostly); however, the second‐order questions—‘For whom?’ ‘For how long?’ ‘At what cost?’ ‘How can it be optimised?’—have not yet been systematically answered.

### 5.2 Methodological Assessment of Evidence Quality

**Figure 5:**
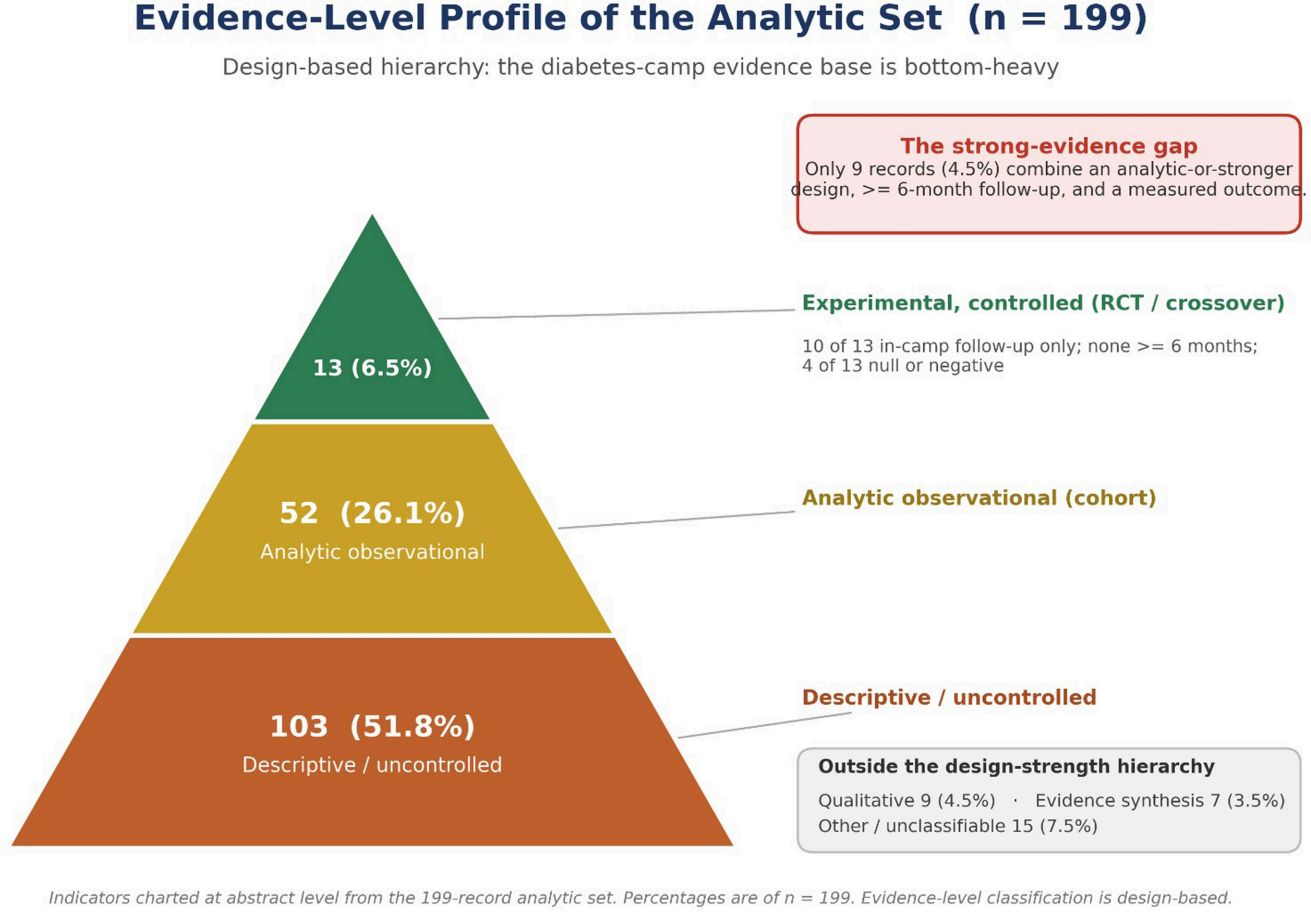
Evidence‐level profile of the 199‐record analytic set. Descriptive or uncontrolled designs constitute the base (*n* = 103, 51.8%); analytic observational designs (prospective or retrospective cohort) the middle tier (*n* = 52, 26.1%), and experimental controlled designs (RCT or crossover) the apex (*n* = 13, 6.5%). Qualitative studies (*n* = 9), evidence syntheses (*n* = 7) and other or unclassifiable records (*n* = 15) fall outside the design‐strength hierarchy. Only 9 records (4.5%) combine an analytic‐or‐stronger design, follow‐up of at least 6 months and a measured outcome.

This review maps studies at heterogeneous evidence levels, which is itself a key finding. In the 199‐record analytic set, only 6.5% (*n* = 13) employed an experimental controlled design (RCT or crossover), 26.1% (*n* = 52) were analytic observational (prospective or retrospective cohort) and 51.8% (*n* = 103) were descriptive or uncontrolled; qualitative studies, evidence syntheses and other designs made up the remainder. Durable follow‐up is rare (6.0% report at least 6 months), and just over half (52.3%) report a measured directional outcome. Critically, only 9 records (4.5%) combine an analytic‐or‐stronger design, follow‐up of at least 6 months and a measured outcome (Table 2; Figure 5). Even among the 13 controlled studies, follow‐up is predominantly in‐camp (10 of 13), none extends to 6 months, and four report a null or negative result. The predominantly positive picture therefore rests largely on studies of low‐to‐moderate methodological rigour, while high‐quality experimental evidence remains scarce; readers should bear in mind that not all referenced evidence carries equal weight. Table 2 should be read as follows: for each coded dimension, the number and percentage of records in each category are reported as *n* (%), and the two final columns give the lower confidence limit (LCL) and the upper confidence limit (UCL) of the Wilson 95% confidence interval for that percentage. The width of the LCL–UCL range conveys the precision of each estimate directly: the proportion of records with a measured directional outcome, 52.3% (LCL = 45.3; UCL = 59.1), is estimated with substantially greater precision than the proportion of negative or null results among outcome‐reporting records, 9.6% (LCL = 5.3; UCL = 16.8), which rests on only 10 records. Even the most generous reading of the composite rigour indicator, 4.5% (LCL = 2.4; UCL = 8.4), leaves fewer than one record in 10 satisfying all three quality criteria simultaneously, and the UCL of 1.9 for direct cost‐effectiveness evidence shows that, even after allowing for sampling uncertainty, such evidence cannot plausibly exceed 2% of the analytic set.

**Table 2:** Methodological characterisation of the 199‐record analytic set: Distribution across the coded dimensions, reported as *n* (%) with the lower confidence limit (LCL) and upper confidence limit (UCL) of the Wilson 95% confidence interval.

Dimension	Category	*n* (%)	LCL	UCL
Outcome direction (*n* = 199)	Measured directional outcome	104 (52.3)	45.3	59.1
	No measured directional outcome	95 (47.7)	40.9	54.7
Outcome direction, outcome‐reporting records (*n* = 104)	Entirely positive	60 (57.7)	48.1	66.7
	Mixed	34 (32.7)	24.4	42.2
	Negative/null	10 (9.6)	5.3	16.8
Study design (*n* = 199)	RCT or crossover	13 (6.5)	3.9	10.9
	Analytic observational (prospective or retrospective cohort)	52 (26.1)	20.5	32.6
	Descriptive or uncontrolled	103 (51.8)	44.8	58.6
	Qualitative	9 (4.5)	2.4	8.4
	Evidence synthesis	7 (3.5)	1.7	7.1
	Other/unclassifiable	15 (7.5)	4.6	12.1
Follow‐up duration (*n* = 199)	In‐camp (≤ 1 week)	69 (34.7)	28.4	41.5
	Short (1–4 weeks)	8 (4.0)	2.1	7.7
	Medium (1–6 months)	17 (8.5)	5.4	13.3
	Long (≥ 6 months)	12 (6.0)	3.5	10.2
	Not stated	93 (46.7)	39.9	53.7
Composite indicators (*n* = 199)	Analytic‐or‐stronger design + ≥ 6‐month follow‐up + measured outcome	9 (4.5)	2.4	8.4
	Direct cost‐effectiveness evidence	0 (0.0)	0.0	1.9

*Note:* Values are presented as *n* (%). LCL and UCL of the Wilson 95% score confidence interval for the corresponding percentage. Percentages for the outcome‐direction subcategories are calculated over the 104 outcome‐reporting records; all other percentages are calculated over the 199‐record analytic set.

A sensitivity analysis was conducted to test the robustness of the headline figures. Under the current (liberal) definition, a record is classified as positive if it reports significant improvement in at least one dimension; under a stricter definition requiring significance in all reported primary outcomes, the entirely positive subset (57.7%, *n* = 60) contracts while the mixed category expands correspondingly. The benefit‐in‐at‐least‐one‐dimension figure (90.4%) combines the positive and mixed categories and is therefore unchanged by the stricter rule. The shift reinforces the paradox: the majority of outcome‐reporting records show selective rather than comprehensive benefit.

### 5.3 Original Contribution

The fundamental original contribution of this study can be expressed as follows: the diabetes camp literature is a structurally incomplete evidence field that documents short‐term positive effects but inadequately answers questions of sustainability, access equity, cost and delivery models. This incompleteness does not indicate that camps are ineffective, but rather that their effect is neither unconditional nor universal. In the words of Barone et al. [4], ‘expected improvements were not found in all studies’; this study maps the structural reasons underlying that statement.

The framing of this review is deliberately descriptive: it maps the extent and distribution of the evidence and identifies where the coded record is empty, rather than arguing that camps should be funded or delivered in a particular way. To keep the interpretation grounded in the data, every charted proportion is reported with a Wilson 95% confidence interval and every gap is tied to an explicit matrix rule (Section 4.7 that the same gaps can be located independently of the authors' reading of the literature.

### 5.4 Limitations

The main limitations of this study are as follows. First, charting of the analytic set was conducted at abstract level rather than by full‐text appraisal; the 199 analytic records all carry an extractable abstract, while records without an extractable abstract were excluded from the analytic set rather than coded for outcome direction. Second, formal critical appraisal is optional in scoping reviews and was not performed as a per‐study risk‐of‐bias score; instead, a structured methodological characterisation of the analytic set is reported (Section 6.2). Third, although four databases were searched (Web of Science, Scopus, OVID/MEDLINE and PubMed), Embase and CINAHL were not included; the omission of CINAHL is particularly relevant for nursing audiences and may have understated the visibility of nurse‐authored camp scholarship. Fourth, an English‐language restriction was applied, and 107 non‐English records across 17 languages were excluded, which may have omitted relevant non‐English evidence. Fifth, this scoping review was not prospectively registered in a public protocol database beyond the repository deposit.

A further interpretive limitation is publication bias. Only 9.6% of the outcome‐reporting records in the analytic set reported entirely negative or null results, a figure that almost certainly understates the true proportion of null findings. Publication bias systematically favours positive findings: studies demonstrating effectiveness are more likely to be submitted and accepted, while null or negative results remain unpublished in what is known as the file drawer. The relative scarcity of negative findings in this literature may therefore reflect the selective visibility of confirmatory research rather than the true effectiveness of diabetes camps. Any synthesis of this evidence base should account for this structural optimism.

### 5.5 Implications for Nursing Practice and Research

The evidence gaps documented in this study translate into five sets of nursing‐actionable recommendations.

#### 5.5.1 For Nurses Delivering Camp Care

Integrate standardised pre‐post measurement batteries covering metabolic, psychosocial and self‐management domains, and build 6‐month follow‐up data collection into camp programme design—converting episodic camp encounters into longitudinal nurse‐led monitoring relationships.

#### 5.5.2 For Nurse Educators

Use the 32.7% mixed‐outcome finding as a teaching case in evidence‐based practice modules, illustrating how headline success figures can mask sub‐outcome variability and shaping critical‐appraisal skills in undergraduate and postgraduate nursing curricula.

#### 5.5.3 For Nursing Researchers

Prioritise pragmatic randomised designs comparing physical and virtual nurse‐led delivery models with follow‐up of at least 12 months, given that 51.8% of the existing literature is descriptive and short‐term.

#### 5.5.4 For Nursing Professional Associations (International Council of Nurses, Royal College of Nursing and Association of Diabetes Care & Education Specialists)

Update programme guidance to acknowledge the absence of cost‐effectiveness data, the consistent post‐camp deterioration pattern [27] and the lack of universal nurse‐led operational guidelines [42].

#### 5.5.5 For Nurse Leaders, Funders and Policy Makers

Treat the structural absence of economic evidence as a barrier to rational nursing‐workforce allocation in camp‐based programmes; require cost‐effectiveness reporting and budget transparency as conditions of camp programme grants.

## 6 Conclusion

This study revealed the Diabetes Camp Paradox through systematic analysis of a four‐database evidence map of 279 records (199 with extractable abstracts) across five epistemic layers and presented an operationalised evidence gap matrix. The literature demonstrates that camps are mostly beneficial (90.4% of outcome‐reporting records positive in at least one dimension); yet this benefit is selective (32.7% mixed), temporally fragile (consistent post‐camp deterioration and only 6.0% with 6‐month follow‐up), socially inequitable (socioeconomic and ethnic access disparities) and its cost is entirely unknown (zero cost‐effectiveness studies).

Resolving the paradox requires three strategic transformations: temporal expansion (transitioning from during‐camp measurements to follow‐up designs of at least 6–12 months and integration of post‐camp digital reinforcement programmes), dimensional expansion (transitioning from unidimensional outcome measures to multidimensional packages) and structural expansion (transitioning from clinical‐outcome‐focused studies to cost‐effectiveness analyses, universal operational guidelines and comparative delivery‐model assessments). For nursing—the discipline that has historically delivered, coordinated and led these interventions—bridging this evidence gap is not a methodological luxury but a precondition for accountable, equitable and economically defensible nurse‐led care. These transformations will enable the transition from ‘knowing that diabetes camps work’ to ‘knowing how, for whom and at what cost they work’.

## Author Contributions

**M. Nurullah Kurutkan:** methodology, software, formal analysis, data curation, writing – original draft. **Ilknur Arslanoğlu:** conceptualization, investigation, validation, supervision, writing – review and editing.

## Funding

The authors have nothing to report.

## Ethics Statement

The authors have nothing to report.

## Conflicts of Interest

The authors declare no conflicts of interest.

## AI Disclosure

The authors used an AI‐assisted writing tool (Claude, Anthropic) for language editing, abstract restructuring, formatting of end‐matter sections and reference format conversion. The tool was not used for literature searching, screening, data coding, quantitative analysis or interpretation of findings. All analytical and intellectual content is solely the work of the authors.

## Social Media Post

Camps work. But for how long, for whom and at what cost? A scoping review of 279 publications finds zero CEA studies in diabetes camp literature. @DiabetologiaJnl #DiabetesCamp #EvidenceGap #T1D.

## Supporting information


Supporting File 1

Supporting File 2

Supporting File 3

Supporting File 4

## Data Availability

The data that support the findings of this study are available in the supporting information of this article. Protocol DOI: https://doi.org/10.5281/zenodo.19582024 and Dataset DOI: https://zenodo.org/records/19581692.
