# Supplementary material for: The Diabetes Camp Paradox: An Evidence Gap Map of 279 Publications and the Anatomy of Known Unknowns
Source: Health Expect. 2026 Aug 22;29(4):e70833. doi: 10.1111/hex.70833 (PMC13499012; doi:10.1111/hex.70833)
Supplement: Supplementary file 1 [file HEX-29-e70833-s001.docx]

# Supplementary File S2. Full Per-Database Search Strategies

Manuscript: The Diabetes Camp Paradox: An Evidence Gap Map of 279 Records and the Anatomy of Known Unknowns. All searches were executed on 19 June 2026 without date restriction.

| Database | Search field | Query string | Records retrieved |
| --- | --- | --- | --- |
| Web of Science Core Collection | Title | TI=(diabet* AND camp) | 750 |
| Scopus | Title | TITLE(diabet* AND camp) | 508 |
| OVID/MEDLINE | Title | (diabet* and camp).ti. | 333 |
| Pooled primary databases | — | — | 1,591 |
| PubMed (supplementary arm) | Title | PubMed query string executed on 19 June 2026: title-field 'diabet*' AND 'camp' with query-level exclusion of 'cyclic AMP'/'cAMP' and 'refugee' | 13 new records after de-duplication |

Processing notes. The pooled 1,591 records were screened for lexical relevance against native per-database exports (655 homonym records removed). Cross-database de-duplication used DOI matching plus exact-title matching (376 duplicates removed), leaving 560 unique records. Publication-type filtering retained journal articles, early-access articles and reviews (187 records removed); an English-language restriction removed 107 non-English records across 17 languages, leaving 266 records. The supplementary PubMed search added 13 new records, producing the de-duplicated evidence map of 279 records. Because the unfiltered PubMed title query returned 4,555 records dominated by the cyclic-AMP homonym, PubMed was searched with an a-priori filtered strategy and treated as a supplementary source. Embase was not searched because the authors' institutions hold no Embase subscription; this is acknowledged as a limitation in the manuscript.
