# Supplementary material for: The Diabetes Camp Paradox: An Evidence Gap Map of 279 Publications and the Anatomy of Known Unknowns
Source: Health Expect. 2026 Aug 22;29(4):e70833. doi: 10.1111/hex.70833 (PMC13499012; doi:10.1111/hex.70833)
Supplement: Supplementary file 2 [file HEX-29-e70833-s002.docx]

**PRISMA-ScR Checklist**

*The Diabetes Camp Paradox: An Evidence Gap Map | Mapped to the revised manuscript (Tricco et al., 2018). Items 12 and 16 (critical appraisal) are optional in scoping reviews.*

| **Item** | **Opt.** | **Checklist item** | **Reported in revision (location)** |
| --- | --- | --- | --- |
| **TITLE** | | | |
| **1** |  | Identify the report as a scoping review. | Title page (title states scoping review) |
| **ABSTRACT** | | | |
| **2** |  | Structured summary (background, objectives, eligibility, sources, charting methods, results, conclusions). | Abstract |
| **INTRODUCTION** | | | |
| **3** |  | Describe the rationale for the review. | Introduction |
| **4** |  | Provide an explicit statement of the questions and objectives. | Introduction (aim and review questions) |
| **METHODS** | | | |
| **5** |  | Indicate whether a protocol exists and where it can be accessed; registration. | Methods - Protocol (protocol available from authors; registration status stated) |
| **6** |  | Specify the eligibility criteria for sources of evidence. | Methods - Eligibility; three-tier criteria (evidence map 279 / analytic set 199) |
| **7** |  | Describe all information sources and the date last searched. | Methods - Information sources; four databases (WoS, Scopus, OVID/MEDLINE, PubMed); last searched 19 June 2026; search-strategy table |
| **8** |  | Present the full electronic search strategy for at least one database. | Methods - Search; per-database strategies; PubMed strategy verbatim (search-strategy table) |
| **9** |  | State the process for selecting sources of evidence. | Methods - Study selection; Figure 1 (PRISMA-ScR flow); dual-author screening and adjudication |
| **10** |  | Describe the methods of charting data (independent/duplicate; confirmation). | Methods - Data charting; directed content analysis; two dimensions double-coded blind; IRR (kappa 0.955 / 0.960) |
| **11** |  | List and define all variables data were sought for; assumptions/simplifications. | Methods - Data items; codebook (four dimensions: outcome direction, design, follow-up, theme) |
| **12** | opt. | Critical appraisal of individual sources of evidence (if done): rationale, methods, use. | Methods - Quality characterization; appraisal optional in scoping reviews; design-based methodological profile (Table 2; Section 5.2) |
| **13** |  | Describe the methods of handling and summarizing the charted data. | Methods - Synthesis; descriptive mapping; proportions with Wilson 95% CIs (Table 2) |
| **RESULTS** | | | |
| **14** |  | Give numbers screened, assessed, and included, with exclusion reasons (flow diagram). | Results; Figure 1 (1,591 to 266 to 279 to 199 with itemized exclusions) |
| **15** |  | Present the characteristics for which data were charted, with citations. | Results; characteristics summary; analytic dataset (199 records, provenance included) |
| **16** | opt. | Critical appraisal within sources of evidence (if done): present data. | Results/Discussion - methodological profile (Table 2; Figure 5) |
| **17** |  | For each source, present the charted data relevant to the review questions. | Results; per-record charting in the supplementary analytic dataset (199) |
| **18** |  | Summarize and present the charting results relative to the questions/objectives. | Results; distributions of outcome direction, design, follow-up, theme; Venn (database provenance); evidence pyramid |
| **DISCUSSION** | | | |
| **19** |  | Summarize the main results (concepts, themes, types of evidence); link to questions. | Discussion - summary of evidence |
| **20** |  | Discuss the limitations of the scoping review process. | Discussion - limitations (Embase not searched; abstract-level charting; English restriction) |
| **21** |  | Provide a general interpretation; implications and next steps. | Discussion - conclusions and implications |
| **FUNDING** | | | |
| **22** |  | Describe funding for included sources and for the scoping review; funder role. | Funding statement |

*Reference: Tricco, A. C., et al. (2018). PRISMA Extension for Scoping Reviews (PRISMA-ScR). Annals of Internal Medicine, 169(7), 467-473. https://doi.org/10.7326/M18-0850*
