# Supplementary material for: The Diabetes Camp Paradox: An Evidence Gap Map of 279 Publications and the Anatomy of Known Unknowns
Source: Health Expect. 2026 Aug 22;29(4):e70833. doi: 10.1111/hex.70833 (PMC13499012; doi:10.1111/hex.70833)
Supplement: Supplementary file 3 [file HEX-29-e70833-s003.docx]

**Coding Protocol, Inter-Rater Reliability, Impartiality Safeguards, and Quality Characterization**

*The Diabetes Camp Paradox: An Evidence Gap Map | Methods revision and response to Reviewer 1 (items 3, 4, 6)*

# **A. Data charting and coding protocol**

The 199 abstract-bearing records of the analytic set were charted using directed (deductive-dominant) qualitative content analysis (Hsieh & Shannon, 2005). A structured codebook was specified a priori from the review's conceptual framework and refined inductively during a calibration round on a pilot subset before full coding began. Four dimensions were charted, each record being assigned exactly one category per dimension on the basis of its title and abstract, with a free-text rationale recorded for every coding decision to support auditability (Table 1). The two interpretively-demanding dimensions, outcome direction and study design, were independently double-coded; follow-up duration and thematic area were charted by one reviewer and verified against the abstract by a second.

**Table 1. Charting codebook**

| **Dimension** | **Categories** |
| --- | --- |
| **Outcome direction** | Positive; Mixed; Negative/Null; No outcome reported |
| **Study design** | RCT/crossover; Prospective cohort; Retrospective; Descriptive; Qualitative; Pilot/feasibility; Narrative review; Systematic review/Meta-analysis; Other |
| **Follow-up duration** | In-camp (<=1 week); Short (1-4 weeks); Medium (1-6 months); Long (>=6 months); Not stated |
| **Thematic area** | Metabolic/glycaemic; Psychosocial; Technology; Education/self-management; Organizational/safety; Financial; Professional learning; Access/equity; Other |

# **B. Inter-rater reliability**

Outcome direction and study design were independently coded by two reviewers blinded to one another's codes. Agreement was quantified with Cohen's kappa (Cohen, 1960). To avoid spuriously inflating agreement, records that either coder judged out of scope were retained in the kappa computation as a distinct category rather than removed; kappa was therefore computed on the full set of 207 double-coded records. Agreement was almost perfect for both dimensions (Table 2). All disagreements (two substantive outcome-direction disagreements, two substantive study-design disagreements, and the scope and data-integrity discrepancies) were resolved by consensus discussion and recorded in an adjudication log retained with the analytic dataset.

**Table 2. Inter-rater reliability (207 double-coded records)**

| **Dimension** | **Cohen kappa** | **95% CI** | **Agreement** |
| --- | --- | --- | --- |
| Outcome direction | **0.955** | 0.920 - 0.991 | Almost perfect |
| Study design | **0.960** | 0.929 - 0.992 | Almost perfect |

*Note. Interpretation follows Landis and Koch (1977). Confidence intervals are large-sample (Wald) intervals, upper-bounded at 1.00.*

# **C. Safeguards against confirmation bias**

Because the review advances a paradox interpretation, several procedural safeguards were built in to protect against confirmation bias.

1. The codebook was specified before coding and applied uniformly; categories were not adjusted to favour the paradox interpretation.
2. The two interpretively-demanding dimensions were double-coded blind; the near-perfect agreement (kappa >= 0.955) indicates the coding is reproducible rather than idiosyncratic.
3. The outcome-direction scheme is balanced (Positive, Mixed, Negative/Null, No outcome reported); the observed distribution emerges from the data, not from a scheme designed to detect a paradox.
4. Where coders differed on ambiguous or qualitative records, the consensus rule deliberately coded toward ‘no measured outcome’ rather than ‘positive’, so that favourable but unmeasured impressions were not counted as positive evidence. This rule is conservative against, not toward, the paradox.
5. No record was excluded on the basis of the direction of its result.
6. Scope exclusions were made by dual-author full-text review against pre-stated criteria and are itemized in the adjudication log.
7. The analytic dataset, the database-provenance coding, and the adjudication log are provided so that the coding can be independently reproduced.

# **D. Quality and methodological characterization of the evidence base**

## **D.1 Rationale**

A formal critical appraisal or risk-of-bias assessment of individual sources is not a required component of a scoping review. The defining purpose of a scoping review is to map the extent, range, and nature of the evidence rather than to pool or grade effect estimates (Arksey & O'Malley, 2005; Levac et al., 2010; Munn et al., 2018; Peters et al., 2020; Tricco et al., 2018). Per-study risk-of-bias scores were therefore not assigned. A full-text appraisal instrument such as the Mixed Methods Appraisal Tool (Hong et al., 2018) further requires the full text of every study across multiple design-specific criteria, which falls outside the abstract-level mapping scope of this review.

## **D.2 Methodological profile of the analytic set**

To address the reviewer's concern transparently and to substantiate the paradox, the methodological profile of the 199 analytic records was characterized using indicators derivable at the abstract level: a design-based evidence-level classification, the presence of a controlled comparison, follow-up duration, and completeness of outcome reporting (Tables 3 and 4).

**Table 3. Design-based evidence level (n = 199)**

| **Evidence level (design)** | **n** | **%** |
| --- | --- | --- |
| Experimental, controlled (RCT/crossover) | **13** | 6.5% |
| Analytic observational (prospective/retrospective cohort) | **52** | 26.1% |
| Descriptive / uncontrolled (descriptive, pilot/feasibility) | **103** | 51.8% |
| Qualitative | **9** | 4.5% |
| Evidence synthesis (narrative; systematic/meta-analysis) | **7** | 3.5% |
| Other / unclassifiable | **15** | 7.5% |
| **Total** | **199** | 100% |

**Table 4. Reporting-quality indicators (n = 199)**

| **Indicator** | **n** | **%** |
| --- | --- | --- |
| Controlled comparison present (experimental design) | **13** | 6.5% |
| Follow-up >= 6 months | **12** | 6.0% |
| In-camp observation only (<= 1 week) | **69** | 34.7% |
| Follow-up not stated | **93** | 46.7% |
| Measured directional outcome reported | **104** | 52.3% |
| **Design (analytic+) AND >= 6 mo follow-up AND measured outcome** | **9** | 4.5% |

## **D.3 Key finding**

The evidence base is dominated by descriptive or uncontrolled designs (51.8%), with only 6.5% employing an experimental controlled design. Durable follow-up is rare: only 6.0% of records report follow-up of at least six months, whereas 34.7% report only in-camp (<= 1 week) observation and 46.7% do not state any follow-up. Just over half (52.3%) report a measured directional outcome. Critically, only nine records (4.5%) combine an analytic-or-stronger design, follow-up of at least six months, and a measured outcome. Even within the 13 controlled (RCT/crossover) studies, follow-up is predominantly in-camp (10 of 13), none extends to six months, and four of thirteen report a null or negative result. This methodological profile is itself the evidence gap that the review maps: the proliferation of diabetes-camp activity is not matched by durable, controlled, outcome-reporting evidence.

# **E. Point-by-point response**

**Item 3 - Absence of formal quality assessment.**

Formal critical appraisal is optional in scoping reviews and is not part of the established methodological framework (Arksey & O'Malley, 2005; Levac et al., 2010; Peters et al., 2020; Tricco et al., 2018). Rather than omit any quality consideration, the revision adds a structured methodological characterization of the 199 analytic records (Tables 3 and 4) showing that the evidence is predominantly descriptive, short-term, and incomplete in outcome reporting, with only 4.5% combining an adequate design, durable follow-up, and a measured outcome. This characterization both respects scoping-review methodology and directly evidences the paradox.

**Item 4 - Coding/extraction protocol not described.**

The revision specifies the directed content-analysis protocol, the a-priori codebook (Table 1), the calibration round, the per-decision rationale, and the double-coding of the two interpretively-demanding dimensions with formal inter-rater reliability (Table 2; outcome direction kappa 0.955, study design kappa 0.960).

**Item 6 - The review may be designed to support the paradox.**

Seven procedural safeguards against confirmation bias are now documented (Section C): a pre-specified uniform codebook, blind double-coding with near-perfect reliability, a balanced outcome scheme, a consensus rule that is conservative against the paradox, no exclusion by result direction, dual-author scope adjudication, and full data and log sharing for reproducibility.

# **References**

Arksey, H., & O'Malley, L. (2005). Scoping studies: Towards a methodological framework. International Journal of Social Research Methodology, 8(1), 19-32. https://doi.org/10.1080/1364557032000119616

Cohen, J. (1960). A coefficient of agreement for nominal scales. Educational and Psychological Measurement, 20(1), 37-46. https://doi.org/10.1177/001316446002000104

Hong, Q. N., Fabregues, S., Bartlett, G., Boardman, F., Cargo, M., Dagenais, P., ... Pluye, P. (2018). The Mixed Methods Appraisal Tool (MMAT) version 2018. Education for Information, 34(4), 285-291. https://doi.org/10.3233/EFI-180221

Hsieh, H.-F., & Shannon, S. E. (2005). Three approaches to qualitative content analysis. Qualitative Health Research, 15(9), 1277-1288. https://doi.org/10.1177/1049732305276687

Landis, J. R., & Koch, G. G. (1977). The measurement of observer agreement for categorical data. Biometrics, 33(1), 159-174. https://doi.org/10.2307/2529310

Levac, D., Colquhoun, H., & O'Brien, K. K. (2010). Scoping studies: Advancing the methodology. Implementation Science, 5, 69. https://doi.org/10.1186/1748-5908-5-69

Munn, Z., Peters, M. D. J., Stern, C., Tufanaru, C., McArthur, A., & Aromataris, E. (2018). Systematic review or scoping review? BMC Medical Research Methodology, 18, 143. https://doi.org/10.1186/s12874-018-0611-x

Peters, M. D. J., Marnie, C., Tricco, A. C., Pollock, D., Munn, Z., Alexander, L., ... Khalil, H. (2020). Updated methodological guidance for the conduct of scoping reviews. JBI Evidence Synthesis, 18(10), 2119-2126. https://doi.org/10.11124/JBIES-20-00167

Tricco, A. C., Lillie, E., Zarin, W., O'Brien, K. K., Colquhoun, H., Levac, D., ... Straus, S. E. (2018). PRISMA Extension for Scoping Reviews (PRISMA-ScR): Checklist and explanation. Annals of Internal Medicine, 169(7), 467-473. https://doi.org/10.7326/M18-0850
